# From environmental adaptation to host survival: Attributes that mediate pathogenicity of *Candida auris*

**DOI:** 10.1080/21505594.2022.2026037

**Published:** 2022-02-10

**Authors:** Stefanie Allert, Daniela Schulz, Philipp Kämmer, Peter Großmann, Thomas Wolf, Sascha Schäuble, Gianni Panagiotou, Sascha Brunke, Bernhard Hube

**Affiliations:** a Department of Microbial Pathogenicity Mechanisms, Leibniz Institute for Natural Product Research and Infection Biology, Hans-Knoell-Institute, Jena, Germany; b Systems Biology and Bioinformatics Unit, Leibniz Institute for Natural Product Research and Infection Biology, Hans-Knoell-Institute, Jena, Germany; c Department of Medicine and State Key Laboratory of Pharmaceutical Biotechnology, University of Hong Kong, Hong Kong, China; d Institute of Microbiology, Friedrich-Schiller-University, Jena, Germany

**Keywords:** *Candida auris*, blood infection, transcriptional profiling, neutrophils, ROS, virulence factor, transporter, cell surface

## Abstract

*Candida* species are a major cause of invasive fungal infections. While *Candida albicans, C. glabrata, C. parapsilosis*, and *C. tropicalis* are the most dominant species causing life-threatening candidiasis, *C. auris* recently emerged as a new species causing invasive infections with high rates of clinical treatment failures. To mimic initial phases of systemic *Candida* infections with dissemination *via* the bloodstream and to elucidate the pathogenic potential of *C. auris*, we used an *ex vivo* whole blood infection model. Similar to other clinically relevant *Candida* spp., *C. auris* is efficiently killed in human blood, but showed characteristic patterns of immune cell association, survival rates, and cytokine induction. Dual-species transcriptional profiling of *C. auris*-infected blood revealed a unique *C. auris* gene expression program during infection, while the host response proofed similar and conserved compared to other *Candida* species. *C. auris*-specific responses included adaptation and survival strategies, such as counteracting oxidative burst of immune cells, but also expression of potential virulence factors, (drug) transporters, and cell surface-associated genes. Despite comparable pathogenicity to other *Candida* species in our model, *C. auris*-specific transcriptional adaptations as well as its increased stress resistance and long-term environmental survival, likely contribute to the high risk of contamination and distribution in a nosocomial setting. Moreover, infections of neutrophils with pre-starved *C. auris* cells suggest that environmental preconditioning can have modulatory effects on the early host interaction. In summary, we present novel insights into *C. auris* pathogenicity, revealing adaptations to human blood and environmental niches distinctive from other *Candida* species.

## Introduction

*Candida* species are among the most prevalent human fungal pathogens worldwide, and represent the fourth most common cause of nosocomial (hospital-acquired) bloodstream infections in the US [1,2]. *C. albicans* is the most common cause of candidiasis, and together with *C. glabrata, C. parapsilosis*, and *C. tropicalis*, accounts for around 90% of *Candida* bloodstream infections [3–5]. Recently a new opportunistic *Candida* species, *C. auris*, has emerged and quickly spread to every permanently inhabited continent [6]. This species is of clinical concern as most clinical isolates appear to be resistant against commonly used antifungal drugs, dramatically limiting therapeutic options [7]. Numerous outbreaks in healthcare facilities have been reported in different regions worldwide, with *C. auris* bloodstream infection-associated mortality rates reaching around 30–60% [8,9]. *C. auris* rapidly emerged simultaneously in at least four different clades, each corresponding to a specific geographical region: South Asian clade I (representative strain: B8441 with reference genome available at the *Candida* Genome Database, CGD [10]), East Asian clade II, African clade III (representative strain: B11221 with sequenced genome on CGD), and South American clade IV [11–13]. The first clinically reported *C. auris* isolate from 2009 belongs to clade II and was derived from an unusual ear infection [14,15]. Recent *C. auris* infections and hospital outbreaks have been associated with strains from clades I, III, and IV [16,17]. In these cases, *C. auris* has been not only isolated from the skin of patients and medical devices, but was also recovered from blood, urine, peritoneal, vaginal, and respiratory samples [9,18]. While *C. albicans* and *C. glabrata* are usually found associated with the human host [19–22], *C. auris* has been often isolated from the skin and the environment, including the hospital setting, suggesting that *C. auris* is well-adapted for survival outside the human body [23–28]. The species closest related to *C. auris* is *C. haemulonii*, which has been isolated from environmental and superficial host niches [9,12,29]. While *C. haemulonii*, similar to *C. auris*, shows a relatively high intrinsic resistance against antifungals, this related species lacks the ability to grow at elevated (host-relevant) temperatures. In contrast to *C. haemulonii* and most environmental fungi, *C. auris* has been shown to grow readily at 37°C, and even up to 42°C [29]. Apart from thermal tolerance, its high salinity tolerance and resistance against other environmental stresses hint toward a remarkable ability of *C. auris* to survive different environmental conditions [30,31]. The virulence factors of *C. auris* are largely unknown or uncharacterized, but many orthologues of virulence-associated *C. albicans* genes can be found in the *C. auris* genome [32]. For example, the chaperone Hsp90 is associated with *C. albicans* morphogenesis and virulence, and its *C. auris* orthologue was found to be involved in growth, morphology, and antifungal drug tolerance [33]. Although different *in vivo* studies using *Galleria* [34], *Drosophila* [35], zebrafish [36], or mice [29,37] clearly underline the ability of *C. auris* to cause invasive infection in animals, little is known about the pathogenicity mechanisms of this emerging fungus.

To disseminate throughout the human body, *Candida* cells have to enter the bloodstream where they face the immune system and nutrient limitations, especially for those micronutrients that are actively sequestered away by the hosts, such as iron [38]. Multiple arms of the innate immune system, including the complement system, monocytes, and neutrophils immediately act against invading *Candida* cells in the bloodstream. Consequently, *Candida* species including *C. albicans, C. glabrata, C. tropicalis*, and *C. parapsilosis* developed survival strategies for confrontations with innate immune cells or components, especially human neutrophils [39–46], monocytes or dendritic cells [47,48], and the complement system [49–51]. One recent study described resistance of *C. auris* to killing by human neutrophils, associated with reduced recruitment and formation of neutrophil extracellular traps (NETs) compared to *C. albicans* [36]. Other studies described the immune response of peripheral blood mononuclear cells (PBMC) to *C. auris* with regard to the pro-inflammatory response [37,52]. While most of these *in vitro* studies focused on one specific, isolated immune cell type, subsequent work has analyzed cellular and transcriptional responses of host and pathogens during bloodstream infections in a complex *ex vivo* whole blood infection model for *C. albicans, C. glabrata, C. tropicalis*, and *C. parapsilosis* [53,54]. In the present study, we use this *ex vivo* whole blood infection model to characterize and compare *C. auris* pathogenicity and its fungal adaptation and survival strategies which allow its subsequent systemic dissemination. Molecular and cellular events during bloodstream infection are characterized for *C. auris* in comparison to the other four investigated *Candida* spp., including transcriptional responses from the fungal and host sides using dual-species RNA sequencing. We use *in vitro* models to elaborate the crucial roles of human neutrophils during blood infection by *C. auris*. Fungal survival under conditions mimicking hospital settings is assayed for *C. auris* in comparison with other *Candida* species, as this seems to be a prerequisite for nosocomial spreading. Furthermore, we explore whether environmental conditions may prime *C. auris* for survival in the host.

In summary, our data suggest novel *C. auris* features that mediate environmental adaptation, host survival, and pathogenicity, which are in part unique or otherwise similar to other *Candida* species. These analyses enable us to integrate *C. auris* into the pathogenic landscape of clinically relevant *Candida* species.

## Material & methods

### Candida *strains and cultivation*

The *Candida auris* strains used in this study have been kindly provided by the Mycotic Diseases Branch, Centers for Disease Control and Prevention (CDC; Atlanta [11]) and *C. auris* 1715 by Neil Gow (University of Exeter [30]. In order to compare the properties of *C. auris* to other *Candida* species on the molecular level, we focused on the *C. auris* genome reference strain B8441, representing the South Asian clade I [11,12]. *C. albicans* strain SC5314 [55], *C. glabrata* ATCC2001, *C. tropicalis* DSM4959, *C. parapsilosis* GA1 [56], and *C. haemulonii* CBS 5149 T were used for comparison. These strains were also used in previous studies by Kämmer *et al*. and Pekmezovic *et al*. for transcriptional profiling [53,57].

*Candida* strains were routinely grown and maintained on YPD agar plates (1% yeast extract, 2% peptone, 2% glucose, 2% agar) at 30°C. For use in experiments, *Candida* cells were cultured overnight in liquid YPD (1% yeast extract, 2% peptone, 2% glucose) at 30°C, shaking at 180 rpm. Cells from overnight cultures were harvested by centrifugation, washed with PBS, and the cell number was adjusted to the desired concentration as indicated for each experiment.

### Analysis of fungal growth

Fungal growth was analyzed in 96-well plates in YPD and other media as mentioned. *Candida* strains were added at a final density of 2 × 10^6^ cells/well, and growth was determined by measuring the absorbance at 600 nm every 30 min for the indicated time period and temperature in a microplate reader (Tecan). Preparation of mouse organ homogenates was done according to Dunker *et al*. with stepwise filtering of homogenized liver or kidney from male C57BL/6 J mice for final organ concentrations of 0.025 g organs/ml [58].

### Ex vivo *whole blood infection model*

#### Ethics approval and consent to participate

Human peripheral blood was collected from healthy volunteers with written, informed consent. This study was conducted according to the principles expressed in the Declaration of Helsinki. The blood donation protocol and use of blood for this study were approved by the institutional ethics committee of the University Hospital Jena (permission number 2207–01/08).

#### Quantification of fungal survival

Human whole blood was freshly drawn from healthy volunteers and anticoagulated with recombinant Hirudin (Sarstedt). *Candida* cells were harvested as described in `*Candida* strains and cultivation`. *Candida* cells diluted in PBS (Phosphate buffered saline) were added to the blood at a concentration of 1 × 10^6^ cells per ml and further incubated at 37°C. Samples were taken at indicated time points and plated on YPD in appropriate dilutions (in PBS) to determine colony forming units (CFUs) as a measure of fungal survival. These plates were incubated at 30°C to prevent side effects (cell-cell aggregations) due to *C. albicans* filamentation. Blood smears of *Candida*-infected samples were prepared at indicated time points and stained with May-Grünwald-Giemsa staining, dried and visualized microscopically for histological analysis.

#### Determination of immune cell association

Fungal cells from overnight cultures were stained with FITC (fluorescein isothiocyanate), added to freshly drawn human blood at a concentration of 1 × 10^6^ cells per ml and incubated at 37°C. As in previous studies [53,59], samples from infected whole blood were taken at the indicated time points, specific conjugated antibodies were added (see below), treated with FACS Lysing Solution (BD), washed, and immediately analyzed with the BD FACSCanto II flow cytometer counting 100,000 events per sample. The gating strategy is based on plotting forward (FSC) and side scatter (SSC) to exclude cellular debris, followed by gating for single cells using FSC-W and FSC-H. The population of single cells was further plotted by using SSC-A and CD445 to get the population of leukocytes as well as by using FITC-A and SSC-A to get leukocytes associated with fungal cells. To distinguish different immune cells in both of these leukocyte populations, specific staining with the following conjugated antibodies was used CD45-PE-Cy7 (clone HI30, leukocytes), mouse anti-human CD3-PerCP (clone SK7, T cells), CD19-PE (clone HIB19, B cells), CD56-V450 (clone B159, NK cells), and CD66b-PE (clone G10F5, PMN) obtained from BioLegend. Monocytes were stained with mouse anti-human CD14-PerCP (clone 47–3D6) from Abcam. The presence of activation markers was determined with mouse anti-human CD11b-V450 (clone ICRF44) from BD and CD16-BV510 (clone 3G8), CD69-APC (clone FN50) from BioLegend. For raw data analysis, the software FlowJo v10.0.8 was used.

#### RNA isolation

At indicated time points, infected blood samples were split for the separate isolation of fungal and human RNA. To isolate human RNA, blood aliquots were added to PAXgene Blood RNA Tubes (PreAnalytiX) and processed with the PAXgene Blood RNA Kit (PreAnalytiX) according to the manufacturer’s instructions. To isolate fungal RNA, aliquots of infected blood were added to ice-cold water to lyse human cells, centrifuged, and immediately frozen in liquid nitrogen. The (fungal) cell pellet was further processed with the RiboPure-Yeast Kit (Thermo Fisher Scientific) according to the manufacturer’s instructions. RNA quantity was determined with a NanoDrop 1000 Spectrophotometer (Thermo Fisher Scientific), and RNA quality was verified with the help of an Agilent 2100 Bioanalyzer (Agilent Technologies). Fungal and human RNA samples were pooled subsequently in a quantitative ratio of 1:6 taking different genome sizes into account. All samples were prepared in three biological replicates with independent donors.

#### RNA sequencing

Library preparation and RNA sequencing were carried out at Eurofins Genomics GmbH (Ebersberg, Germany) using the Illumina HiSeq 2500 platform. After poly(A) enrichment, mRNA was fragmented, and cDNA libraries were generated for each sample.

All preprocessing was done using the GEO2RNAseq pipeline (v0.100.1 [60]). The raw reads were quality trimmed with Trimmomatic v0.39, rRNA depleted with SortMeRNA v2.1b and quality controlled with FastQC v0.11.8. The *Homo sapiens* genome GRCh38.p13 (release 100.38) and annotation were downloaded from the ENSEMBL database. *C. albicans* SC5314 assembly 22 (s07-m01-r100), *C. auris* B8441 (s01-m01-r05), *C. glabrata* CBS138 (s02-m07-r43), *C. parapsilosis* CDC317 (s01-m03-r41), and *C. tropicalis* MYA-3404 (version from 11 December 2013) genomes and corresponding genome annotations were downloaded from the *Candida* Genome Database (CGD). Sequencing reads were mapped against concatenated genomes comprised of *H. sapiens* and each *Candida* species using HiSat2 v2.1.0. For *H. sapiens* with *C. albicans* all reads that mapped equally well to both species were removed. This was necessary for correct downstream analysis with the diploid *C. albicans* reference genome. Transcriptome coverage was calculated as mapped reads multiplied by read length and divided by transcriptome length. featureCounts v1.34.0 was applied to count the number of uniquely mapped reads within annotated genes. For *C. albicans* with its diploid reference genome we counted uniquely and multimapped reads and merged counts across alleles by averaging. Human and pathogen genes were tested individually for significant differential expression. For further analysis we normalized all transcriptome data by applying Median Ratio Normalization (MRN) for each species separately. For each individual *Candida* species, we computed the log2(fold change) of each time point against the 0 mpi reference time point and thus normalized to species-specific base transcript levels. DESeq2 was used to calculate adjusted p-values based on count values. Afterward the following cutoffs were applied: adjusted p-value ≤ 0.01, abs(log_2_FC) ≥ 1.5 and MRN ≥ 1 for at least one time point. The generated RNA sequencing dataset analyzed in the present study has been deposited in NCBI’s Gene Expression Omnibus [61] under the GEO record GSE179000. The RNA-seq data set for *C. albicans, C. glabrata, C. tropicalis*, and *C. parapsilosis*, originally generated in the study by Kämmer *et al*., had been deposited in NCBI’s Gene Expression Omnibus under the GEO record GSE114180 [53].

#### Gene expression data analyses

The PCA biplot of MRN values of *H. sapiens* is based on all genes, and the PCA biplot of MRN values for the combined *Candida* species is based on all genes that are orthologous among all five *Candida* species. The PCA biplot of log_2_FC values of *H. sapiens* is based on all *H. sapiens* genes with an adjusted p-value ≤0.01 in at least one comparison, the one for the combined *Candida* species is based on all genes that are orthologues among all five *Candida* species and have an adjusted p-value ≤0.01 in at least one comparison. Orthologous genes between *C. albicans, C. auris, C. glabrata*, and *C. parapsilosis* and additionally to *S. cerevisiae* were mapped with orthology information from CGD. Since orthology information for *C. tropicalis* to the other four *Candida* spp. was not available in CGD, information for orthologous genes between *C. tropicalis* and *S. cerevisiae* was retrieved from the *Candida* Gene Order Browser (CGOB). Orthologous genes of *C. tropicalis* to the other four *Candida* spp. were subsequently mapped *via* identical gene names for *S. cerevisiae*.

Gene set enrichment analysis (GSEA 4.1.0 [62]) was performed on the human MRN data sets (diff_of_classes method based on log2FC values *vs* 0 mpi), after conversion to Entrez identifier based on information downloaded from Ensembl Biomart [63], using the Biocarta gene sets of the MSigDB (C2). Gene sets with a false discovery rate q < 0.05 were considered significantly enriched, and their normalized enrichment scores (NES) were plotted for all species and time points. Plots were generated using R 4.1.2 [64] with ggplot2 [65].

#### Quantification of cytokine levels

Infected blood samples taken at indicated time points were centrifuged to obtain plasma and immediately frozen in liquid nitrogen. Concentrations of the cytokines IL-1β, IL-6, IL-8, and TNF-α were determined by ELISA kits according to the manufacturer’s instructions (eBioscience). Cytokine levels were calculated from standard dilutions of the respective recombinant cytokines.

### Infection of neutrophil granulocytes

#### Preparation of neutrophils and serum from human whole blood

Neutrophils were isolated from freshly drawn, whole blood of healthy volunteers using a gradient centrifugation method. Briefly, 1:1 PBS-diluted blood was layered on top of Lymphocyte separation medium (Capricorn) with Ficoll density gradient and centrifuged for 30 min at 700 g. After this, the polymorphonuclear cell (PMN) fraction on top of the erythrocytes was kept, while plasma and the other (cell) layers were removed. Residual erythrocytes were removed by 10–15 min incubation in a hypotonic lysing buffer on ice with subsequent centrifugation for 10 min 1400 rpm at 4°C. This was followed by two analogous washing steps with cold PBS. The resulting cell pellets were resuspended in RPMI1640 w/o phenol red + 10% autologous human serum at a concentration of 5 × 10^5^ cells/ml. For the autologous human serum, blood from the same donors as the PMN isolation was drawn in serum tubes (Sarstedt) and kept for at least 1 h at RT. Subsequently, tubes were centrifuged for 10 min at 2500 g, and the serum was collected for each donor.

#### ROS assay

Neutrophil activation upon stimulation with *Candida* cells was determined by an oxidative burst assay, using the luminol-enhanced chemiluminescence method for the quantification of total reactive oxygen species (ROS). Freshly isolated neutrophils (see above) were seeded at a concentration of 5 × 10^4^ cells/well in a 96 well plate (white, clear bottom, Corning) and incubated for about 1 h at 37°C 5% CO_2_, to let them attach. *Candida* cells, opsonized with human serum for 30 min at 37°C, were washed, and resuspended in RPMI1640 w/o phenol red, and added to neutrophils at 2.5 × 10^5^ cells/well for multiplicity of infection (MOI) 5 or 5 × 10^5^ cells/well for MOI 10. Neutrophils were stimulated with 100 nM phorbol 12-myristate 13-acetate (PMA, Sigma Aldrich) for activation and as positive control. Controls of unstimulated neutrophils and each *Candida* strain without neutrophils were included in each experiment. Luminescence of the *Candida* only control was subtracted from the luminescence of the coincubation. For the detection of total ROS, 50 µl of RPMI1640 containing 200 µM luminol (Sigma Aldrich) and 16 U HRP (Sigma Aldrich) were added to each well, and chemiluminescence was measured every 2.5 min for 2.5 h in a Tecan Infinite M200 microplate reader. Analyses and determination of the area under the curve were performed with GraphPad Prism 8.4.3.

#### Fungal survival assay

Freshly isolated neutrophils (see above) were seeded at a concentration of 5 × 10^4^ cells/well in a 96 well plate and incubated for about 1 h at 37°C 5% CO_2_, to let them attach. *Candida* cells, opsonized with human serum for 30 min at 37°C, were washed and resuspended in RPMI1640 w/o phenol red, and added to neutrophils at 2.5 × 10^4^ cells/well for MOI 0.5 and co-incubated for 1 h or 3 h, respectively. To determine fungal survival, neutrophils were then lysed with 0.02% Triton-X-100 for 5 min. The lysate containing the fungal cells was rigorously collected from the wells, diluted in PBS, and plated on YPD. After 1–2 days of incubation at 30°C, colony forming units (CFUs) were determined. A control of the infectious dose and *Candida* cells without the presence of neutrophils was included for each experiment.

### Statistical analysis

All experiments were conducted in technical duplicates or triplicates (from which the mean value was calculated) on at least three independent occasions (biological replicates). Different biological replicates included blood or neutrophils from different healthy donors. Diagrams show the mean of the biological replicates with standard deviation (SD). Statistical analyses were done with GraphPad Prism 8.

## Results

### *Stress resistance and environmental survival of* Candida auris

In contrast to other pathogenic *Candida* species, *C. auris* is often found in the (healthcare) environment, rather than solely associated with the human host [23,24,27,66]. We propose that adaptation of *C. auris* to environmental stresses allows its survival in a nosocomial setting and may also prime the fungus for survival in certain host niches.

**Figure 1.**
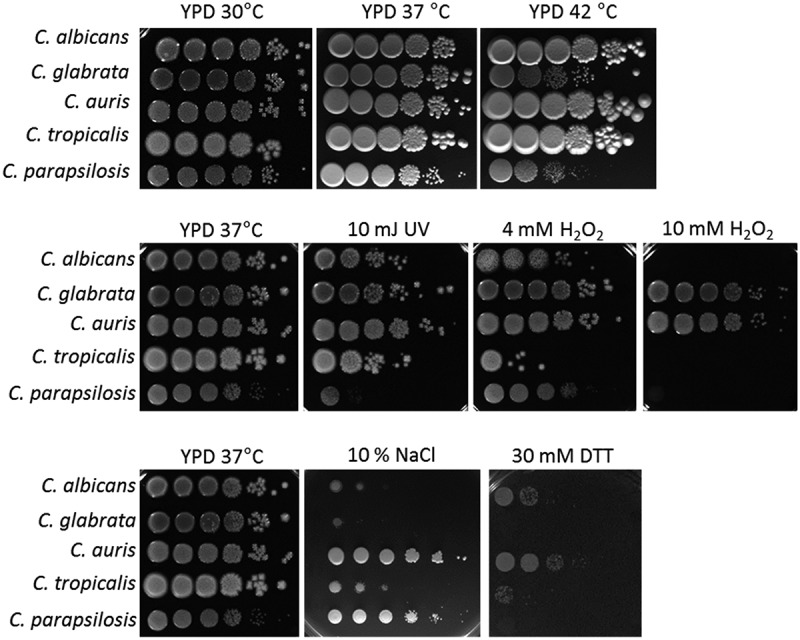
*C. auris* is highly resistant to various stressors *in vitro*. Drop tests with serial dilutions of *C. auris* and the clinically most relevant *Candida* species were performed under various stress conditions to analyze fungal (a) tolerance of oxidative stress, (b) resistance against UV light and high temperature, and (c) resistance against osmotic stress. The highest concentration (left) was 1 × 10^8^ fungal cells/ml. Pictures were taken after 1–2 days of incubation at the indicated temperature and representative examples from at least two biological replicates are shown.

**Figure 2.**
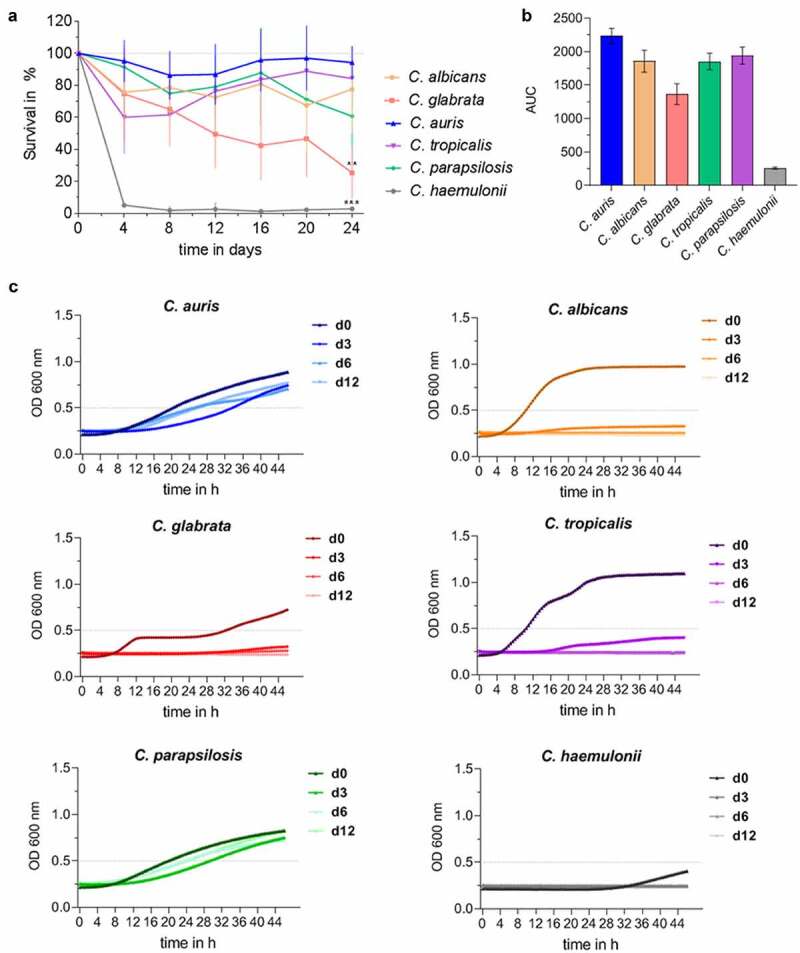
*C. auris* survives long periods under nutrient-limited, environmental conditions. (a) Survival of *Candida* spp. in water at 30°C over a time period of 24 days was analyzed by CFU quantification at indicated time points. Results are shown relative to day 0 as 100%. n ≥ 3, mean ± SD; ** p ≤ 0.005, *** p ≤ 0.001 vs. *C. auris* (b) *Candida s*urvival in water over 24 days from (a) is expressed as Area Under Curve (AUC), clearly indicating differences between *Candida* species. n ≥ 3 (c) Survival of *Candida* spp. after a dry period of 0, 3, 6 or 12 days was assessed by analyzing re-growth in YPD at 30°C by optical density (OD) measurement at 600 nm over 48 h. n ≥ 3 shown as mean.

Using drop tests, we compared the resistance of *C. auris* and the clinically most relevant *Candida* species (*C. albicans, C. glabrata, C. tropicalis*, and *C. parapsilosis*) against oxidative, thermal, and osmotic stress (Figure 1). Among the compared *Candida* species, *C. auris* showed the highest resistance against various stresses, especially growth at 42°C or presence of 10% NaCl. Its growth in the presence of 10 mM H_2_O_2_ was comparable to *C. glabrata*, which is known to have a high tolerance toward oxidative stress (Figure 1 [67]). Another potential stressor in both environmental and host niches is nutrient limitation. To analyze fungal survival under long-term starvation, we incubated *C. auris*, the other four common pathogenic *Candida* species, and the closely related species *C. haemulonii* in distilled water for 24 days. We observed a remarkable ability of *C. auris* to withstand these conditions: About 88% of the originally inoculated fungal population was still able to grow and form colonies after 24 days (Figure 2(a,b)). This was the case for only about 3% and 21% of the original population of *C. haemulonii* and *C. glabrata* cells, respectively. We further analyzed the ability of the different *Candida* species to grow after drying on a plastic surface without nutrient supply (Figure 2(c)). *C. auris* was able to recover and grow following 12 days under such conditions. Only *C. parapsilosis* showed a comparable ability to resist desiccation, while *C. albicans, C. glabrata* and *C. tropicalis* showed no growth after only 3 days. *C. haemulonii* was severely affected by these conditions (Figure 2(c)).

In summary, *C. auris* showed an extremely high level of resistance to a variety of stresses, likely contributing to survival in the nosocomial environmental and therefore to hospital transmissions and the onset of human infections.

### Candida auris *is efficiently killed in human blood, but small subpopulations survive*

**Figure 3.**
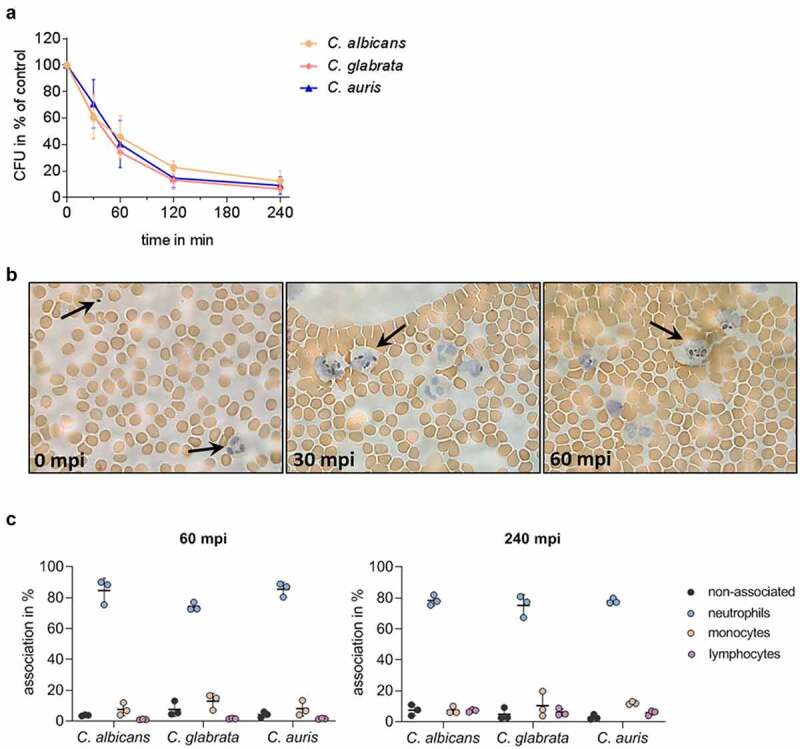
*C. auris* interacts with immune cells and is efficiently killed during *ex vivo* whole blood infection. (a) Survival of *C. auris, C. albicans*, and *C. glabrata* in human whole blood was determined 30, 60, 120, and 240 minutes post infection (mpi) by CFU quantification relative to time point 0 mpi as 100%. n = 6, mean ± SD (b) Exemplary microscopic pictures of blood smears prepared at indicated time points from *C. auris* infected whole blood. Arrows indicate fungal and human immune cells. 100 x magnification (c) Association of fungal cells with immune cells was determined by fluorescence activated cell sorting (FACS) analyses at 60 mpi and 240 mpi. n = 3, mean ± SD for independent experiments using different donors.

During systemic *Candida* infections, fungal cells disseminate *via* the bloodstream throughout the human body. To mimic this critical step of disseminated infections, we applied a previously established *ex vivo* whole-blood infection model [53,54,68]. We found that *C. auris* is efficiently killed in human blood of healthy donors (Figure 3(a), Figure S1A). Within 60 min post infection (mpi), more than 50% of the *C. auris* cells were killed, which is comparable to the killing rates of *C. albicans* and *C. glabrata*. Despite this efficient clearing, however, after 240 mpi a subpopulation of about 9% of *C. auris* cells was found to be still alive (*C. albicans* 12%, *C. glabrata* 6%). Histological analysis of blood smears during the infection showed no aggregation or replication of *C. auris* cells and, in contrast to *C. albicans*, no morphological switch to hyphal growth was detected (Figure 3(b) [53]). Furthermore, we observed co-localization of *C. auris* cells with immune cells. To better characterize which immune cells contribute to killing of *C. auris* cells in the blood, FITC-labeled *C. auris* yeasts and immunofluorescence-stained immune cells were analyzed in a flow cytometry-based assay during their interaction in the *ex vivo* infection model. This analysis showed a rapid association of *C. auris* with blood leukocytes, in particular neutrophils (Figure 3(c)). This is in accordance with observations for other *Candida* spp. like *C. albicans* and *C. glabrata* (Figure 3(c) [53]). After 240 mpi, only 3% of *C. auris* cells were not associated with any immune cell (*C. albicans ≈* 7%, *C. glabrata ≈* 5%). An association with lymphocytes, mainly NK cells, was seen for *C. auris* at the later time point (0.5% 60 mpi; 4.5% 240 mpi; Figure S1B), comparable to lymphocyte associations of *C. albicans* and *C. glabrata*.

Altogether, these observations demonstrate that *C. auris* is efficiently killed in human blood and is mostly associated with neutrophils. However, a fraction of the population remained without contact with any immune cells and/or survived in the *ex vivo* whole-blood infection model.

### *Dual-species transcriptional profiling of* Candida auris*-infected human blood*

To gain more insights into the mechanisms of fungal adaptation to the blood environment, and counteracting human immune response, dual-species transcriptional profiling by RNA sequencing was performed during *C. auris*-whole blood infection. We generated *C. auris* data and used additional-published data sets obtained by the same Standard Operation Procedures for *C. albicans, C. glabrata, C. tropicalis*, and *C. parapsilosis* [53], using MRN values and abs(log_2_FC) ≥ 1.5 (FC > 2.8), adjusted p-value ≤ 0.01 as cutoffs for differential expression of genes.

### *The human transcriptional response to* C. auris *infection resembles the response to other* Candida *species*

**Figure 4.**
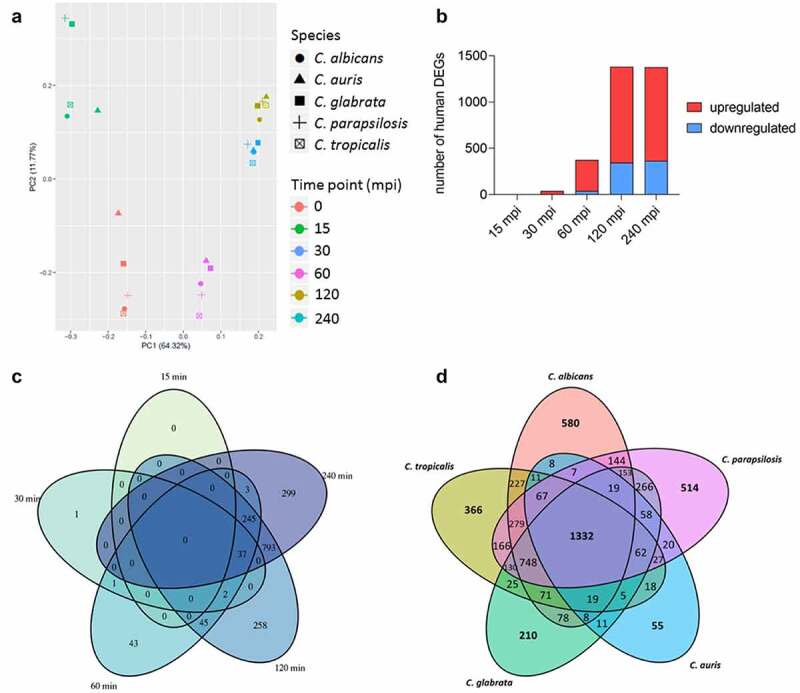
The human transcriptional response to *C. auris* in whole blood infection resembles the response to other *Candida* species. (a) Principal Component analysis (PCA plot) of the human transcriptome based on MRN values. Different time points (color) and *Candida* species (icon) are indicated to show similarity (b) Transcriptional kinetics shown by the number of human up- and down-regulated genes at different time points during *C. auris* infection (compared to 0 min) (c) Venn diagram for *Homo sapiens* comparing differentially expressed genes (DEGs) of each time point (compared to 0 min) during *C. auris* infection. (d) Venn diagram for infection with each indicated *Candida* species comparing human DEGs from infection with the indicated *Candida* species, considering genes differentially expressed during at least one time point.

The transcriptional pattern of human blood cells after infection with each of the five different *Candida* spp. was mainly governed by the time point post infection, while the infecting species had no measurable influence, as seen by Principal Component Analyses (PCA) (Figure 4(a) [53]). After infections with *C. auris*, similar to the other *Candida* spp., the number of regulated human genes was initially very low (Figure 4(b), Figure S2A). Transcriptional responses of blood cells were predominantly visible from 60 min of co-incubation on, with the number of differentially expressed genes (DEGs) increasing to more than 1,000 DEGs (Figure 4(b)). Of about 1,300 DEGs seen at 120 mpi and 240 mpi, 793 genes were differentially regulated exclusively at these two time points (Figure 4(c)), indicating a continuous human response at these later stages of *C. auris* infection.

When comparing transcriptional changes induced by *C. auris* with a published human core response toward infections with *C. albicans, C. glabrata, C. tropicalis*, and *C. parapsilosis* [53] we identified a common and conserved human core response of 1,332 DEGs in total to these four *Candida* spp. and *C. auris* (Figure 2(d), Table S2). In contrast, the number of DEGs specifically regulated in response to any *Candida* species was much lower, with a maximum of 580 DEGs specifically in response to *C. albicans*, and only 55 DEGs specific for the response to *C. auris* (Figure 4(d), Table S2).

**Figure 5.**
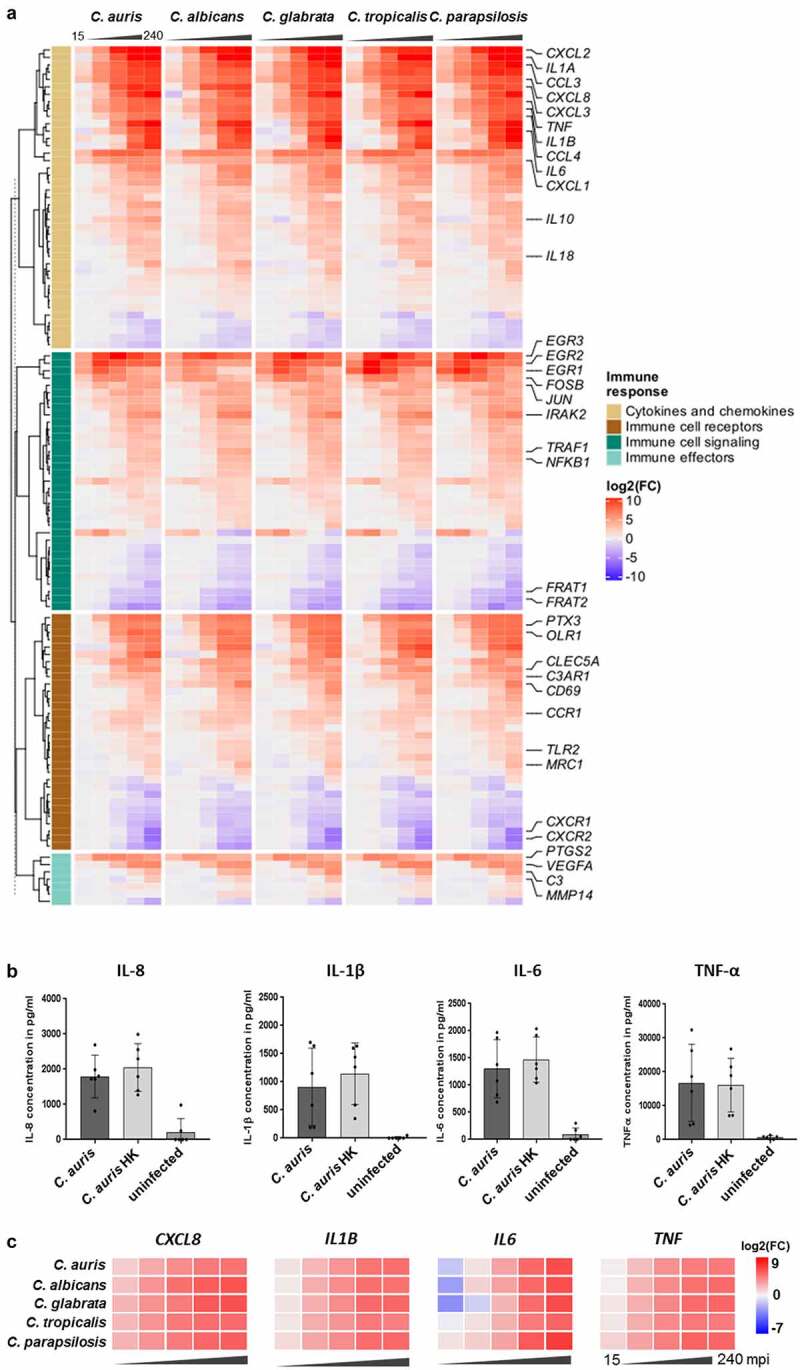
The transcriptional response of blood cells to *C. auris* is dominated by the expression of immune mediators. (a) Expression of human genes encoding immune cell receptors, cytokines or chemokines, or associated with immune cell signaling or effector functions. Gene expression is shown color-coded as log_2_FC vs. 0 min and for each time point (15, 30, 60, 120, 240 mpi). More details are given in Table S3. (b) Plasma levels of the pro-inflammatory cytokines IL-1β, IL-6, TNF-α and the chemokine IL-8 240 mpi with *C. auris* alive or heat-killed (HK) or in an uninfected control sample. n ≥ 5 different donors shown as mean ± SD (c) Expression of selected human cytokines over the time course of *C. auris* infection. Gene expression is shown as log_2_FC vs. 0 min, indicated by color intensity.

The common human response seen for *C. auris* and the other *Candida* species comprises different infection-relevant aspects, including immune recognition, signaling, and immune effector functions (Figure 5(a), Table S3), which is also seen in a gene set enrichment analysis (GSEA) by consistently upregulated inflammatory response (Biocarta LAIR pathway), cytokines (Biocarta CYTOKINE pathway), and IL1R signaling (Biocarta IL1R pathway) sets, among similar others (Figure S2C). Amongst the known fungal-sensing pattern recognition receptors (PRRs [69]), the Toll-like receptor 2 gene (TLR2) was significantly up-regulated during *C. auris* infection (Figure 5(a)), while genes coding for TLR5, TLR6, or TLR8 were rather down-regulated. The genes encoding the C-type lectin receptors (CLR) CLEC5a and MRC1 were significantly up-regulated. Furthermore, for the complement receptor CR3 about 10-fold increased transcript levels were found. In line with the activation of the complement system, the complement activating PRR gene, *PTX3*, as well as the genes encoding the complement component C3 and the complement component receptors C3AR1 and C5AR1 were up-regulated (Figure 5(a)).

Immune recognition *via* PRRs leads to the activation of immunological pathways mediating the production and release of immune effectors and to immune cell activities that counteract invading microbes. Up-regulated genes coding for components known to induce the expression of pro-inflammatory cytokines were EGR transcription factor genes (*EGR1-3*) and genes of the NF-κB pathway, MAPK cascades including JNK, FOS, and AP-1, as well as genes associated with JAK-STAT-signaling (Figure 5(a), Figure S2C [70]). The up-regulation of *NLRP3* during *C. auris* infection further indicates the formation of the NLRP3 inflammasome that leads to the secretion of the pro-inflammatory cytokines IL-1β and IL-18 (*IL1B* > 55-fold up at 60 mpi and > 300-fold up at 240 mpi, *IL18* > 4-fold up at 120 mpi; Figure 5(a), Table S3 [71]). Furthermore, activation of pro-inflammatory immune responses to *C. auris* infections, especially cytokine and chemokine production was indicated by the most highly expressed cytokine gene *IL1A* (> 4,000-fold up at 120–240 mpi), as well as strongly increased expression of *IL6* (> 1,000-fold up), *TNF* (> 170-fold up), and *CXCL8* (encoding IL-8, > 180-fold up, Figure 5(a)), which was also reflected in the gene set enrichment analysis (Figure S2C). The transcriptional up-regulation of these cytokine genes was further confirmed on the protein level for IL-1β, IL-6, TNF-α, and IL-8 at 240 mpi in *C. auris*-infected human blood (Figure 5(b), Figure S1C). The induction of IL-1β, IL-6, TNF-α, and IL-8 was also seen on transcriptional and protein levels as part of the common human core response against other *Candida* spp. (Figure 5(c) [53]). Pro-inflammatory pathways further lead to the recruitment of other immune cells to the site of infection *via* chemokines [72]. In line with this, genes encoding chemokines (e.g. *CCL3, CCL4, CXCL1, CXCL2* and *CXCL3*) and their receptors (*CCR1, CCR5, CXCR2*) were up-regulated in response to *C. auris* ([72,73]; Figure 5(a), Table S3).

Genes encoding the anti-inflammatory cytokines IL-10 [70] and IL1-RA, as well as the TNF-receptor-associated-factor 1 (TRAF1 [74]), that down-regulates the antifungal immune response, were up-regulated only at late time points of *C. auris* blood infection (Figure 5(a)). This might indicate a shift toward a down-regulation of inflammatory responses when most of the fungal cells have been killed (Figure 3(a)). Furthermore, genes encoding matrix metalloproteinases (MMPs) were up-regulated at 240 mpi for all *Candida* species (*mmp14*); specific MMP genes like *mmp9* were up-regulated during *C. auris* and *C. parapsilosis*, but not *C. albicans* infections (Figure 5(a)). MMPs are also known to limit inflammation-induced signals by cytokine inactivation [75,76].

The circulating blood cells orchestrate the immune response by communicating not only with each other but also with endothelial cells. Indeed, we found that genes known to activate endothelial cells were up-regulated in response to *C. auris* (Figure 5(a)), including *PTGS2* (also called *COX2*, encoding the cyclooxygenase producing prostaglandins), a well-described mediator of pro-inflammatory responses [70,77,78]. Furthermore, genes encoding factors that increase vascular permeability and immune cell migration, like TNF-α and IL-1β or the vascular endothelial growth factor (VEGF) were significantly up-regulated in response to *C. auris* (Figure 5(a) [79,80]). The up-regulation of these genes relevant for the modulation of endothelial cells was comparable for *C. auris* and the other four *Candida* species (Figure 5(a)).

In summary, *C. auris* is efficiently recognized by the human immune system during blood infection. Association with neutrophils and monocytes was demonstrated on the cellular level. The transcriptional response by these immune cells showed immune recognition by PRRs. This results in pro-inflammatory activities to counteract the fungal infection, with a large overlap in the gene expression patterns of the host in reaction to infections with *C. auris, C. albicans, C. glabrata, C. tropicalis*, or *C. parapsilosis*. Together with the observed killing of *C. auris* in human blood our data suggest an efficient and controlled immune response against this emerging fungal pathogen. However, a substantial portion of *C. auris* cells can survive 60 min of exposure to human blood and a small fraction was still alive after 240 mpi, indicating fungal adaptation and survival measures.

### *A pattern of its own*: Candida auris *species-specific gene expression during whole blood infection*

**Figure 6.**
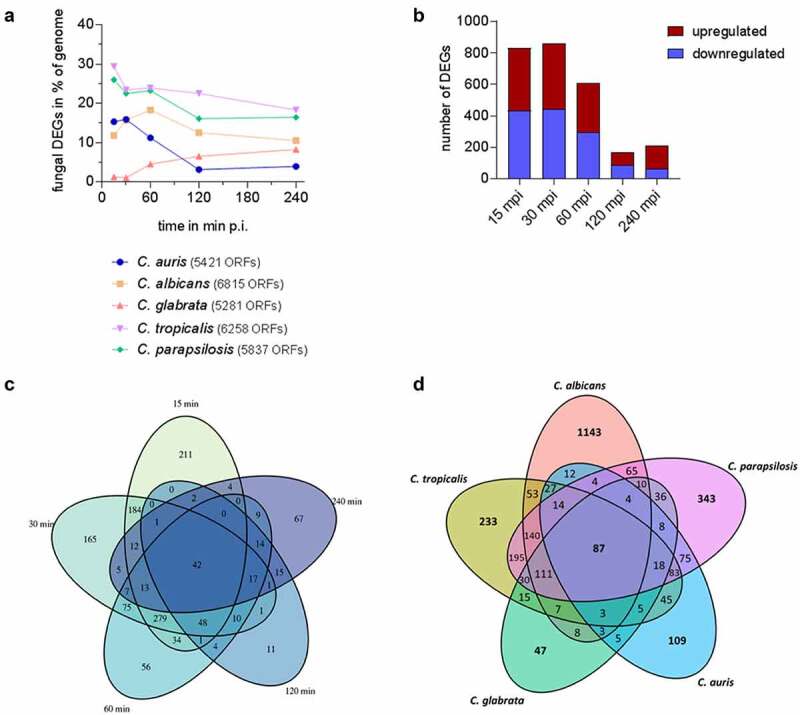
Species-specific gene expression of *C. auris* during whole blood infection. (a) The transcriptional changes during whole blood infection are shown as % of the genome for each *Candida* species at the indicated time points. The total number of predicted open reading frames (ORF) is given in brackets for each species (b) Transcriptional kinetics shown by the number of up- and down-regulated *C. auris* genes at different time points during whole blood infection (compared to 0 min). (c) Venn diagram for *C. auris* comparing fungal differentially expressed genes (DEGs) of each time point (compared to 0 min) during whole blood infection. (d) Venn diagram comparing fungal DEGs for each indicated *Candida* species over the time course of whole blood infection, considering gene orthology and differential expression for at least one time point.

**Figure 7.**
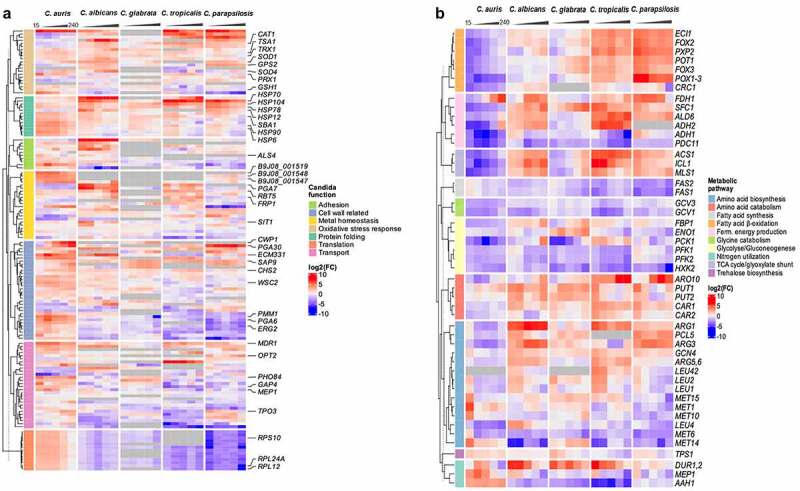
A pattern of its own: *C. auris* specific transcriptional alterations in response to human blood. Expression of selected fungal genes over the course of infection (15, 30, 60, 120, 240 mpi) with the indicated *Candida* species, associated with (a) protein folding, translation, the cell wall, oxidative stress or related to transport across the membrane, metal homeostasis, adhesion, or (b) metabolism. Gene expression is shown for orthologous genes color-coded as log_2_FC vs. 0 min for each time point. Genes without orthologues (or best hit according to BLASTP) are shown in gray. More details are given in Table S6.

Next, we analyzed the transcriptional adaptation of *C. auris* (genome reference strain *C. auris* B8441, representing the South Asian clade I [11,12]) to human blood. It has been previously shown that *C. albicans, C. tropicalis*, and *C. parapsilosis* differentially regulate a significant portion of their genome as early as 15 mpi (Figure 6(a) [53]). For *C. auris*, about 15% of the transcriptome was differentially regulated at early time points, which is more than for *C. glabrata* (1.3–8.2% over the time course, Figure 6(a) [53]). The numbers of genes that were significantly up- and down-regulated in *C. auris* were comparable (Figure 6(b), Figure S2B [53]). The genomes of *C. auris* and the other four *Candida* species share ≈ 3,700 orthologues amongst which only 87 were differentially regulated in all species at any time point during infection (Figure 6(d)). This small set of fungal core response genes includes heat shock protein genes like *HSP104, HSP78*, and *SBA1* which were all up-regulated. They thereby indicate stress adaptation to the hostile blood niche *via* heat shock proteins in all five *Candida* species (Figure 6, Table S5). However, more genes were species-specifically than commonly regulated (Figure 6(d); except for *C. glabrata*). Investigating the time course of *C. auris* DEGs, only 42 DEGs were found to be regulated at all time points (Figure 6(c)). Many of these genes are associated with transmembrane transport, for example *PHO84, HGT10* and *OPT2* (Figure 7(a), Figure S3). The *C. auris* genome contains a substantial number of transporter genes [32,81]. This includes the gene encoding the multidrug resistance efflux pump Mdr1, which is known to play a key role for resistance to azoles in *C. albicans* and also *C. auris* [32,82]. During whole blood infection *MDR1* expression was highly up-regulated at all time points (>12 – 34-fold up), similar to other MDR family members (e.g. B9J08_001319 > 3 – 5-fold up; Figure 7(a), Table S6). Notably, *C. albicans, C. tropicalis*, and *C. parapsilosis* did not up-regulate their orthologous transporter genes to the same extent, and these were also not found to be up-regulated over the whole course of infection.

Furthermore, genes coding for amino acid (*GAP4*) or ammonium transporters (*MEP1*) were up-regulated in *C. auris* (>2 or 8-fold up at 60 mpi, respectively; Figure 7(a)). These transporters may be required for the uptake of essential nutrients and metabolic adaptations. However, the mode of metabolic adaptation seems to differ between *Candida* species. Accordingly, we observed no up-regulation of the glyoxylate cycle genes, *ICL1* and *MLS1*, or genes associated with fermentative energy production (up-regulation of *ADH2 and ALD6*) in *C. auris*, in contrast to *C. albicans* (Figure 7(b)). Furthermore, genes involved in β-oxidation (*CRC1, POX1-3*) were down-regulated in *C. auris* over the entire course of infection, in contrast especially to *C. parapsilosis* (up-regulation of *ECI1, FOX2, FOX3, POT1*, and *PXP2 –* all involved in β-oxidation), indicating differences in the utilization of fatty acids as an energy source (Figure 7(b), Table S6).

While only a small number of *C. auris* genes were differentially expressed at all time points during blood infection, several genes were expressed at more than one time point and the *C. auris* transcriptome can be separated into characteristic early and late responses, with the early 15, 30, and 60 mpi time points having nearly 280 genes in common (Figure 6(c), Table S2). At these early time points, most of the up-regulated genes were involved in translation, like *RPL12, RPL24A*, and *RPS10*, and associated processes such as chaperone-mediated protein folding (Figure S3). While the up-regulation of genes involved in translation was unique for *C. auris* among the tested *Candida* species (Figure 7(a)), transcriptional regulation of protein folding events was commonly found. However, the regulated genes used in these processes were diverse (based on the orthologue data). For example, *HSP6* was solely up-regulated in *C. auris* (> 8.5-fold), while up-regulation of *HSP12* was seen in *C. albicans* and *C. parapsilosis*, but not in *C. auris* (Figure 7(a)). In addition, up-regulation of *HSP90* seems to be relevant for *C. auris* during all stages of blood infection (> 14-fold at early time points; Figure 7(a)), while there was no differential regulation of *HSP90* in *C. albicans* or *C. glabrata. C. albicans, C. tropicalis*, and *C. parapsilosis*, on the other hand, all up-regulated *HSP70* encoding an important Hsp, at least for *C. albicans* virulence [83].

At later time points (120 and 240 mpi), all *Candida* species showed up-regulation of genes associated with cell wall organization and biosynthesis, however, the different species used different sets of genes in this category (Figure 7(a), Figure S3). For example, *CWP1* (encoding Cell Wall Protein 1) is increasingly expressed over the course of infection in *C. auris* (> 470-fold at 240 mpi) and *C. glabrata* (> 13-fold at 240 mpi), while there is no orthologue in *C. albicans*. Similarly, the gene coding for the fungal cell wall protein Pga30 was highly up-regulated in *C. auris* (> 119-fold) and *C. parapsilosis* (> 70-fold), but not in *C. albicans* (Figure 7(a), Table S6). Among the more than 100 DEGs only up-regulated in *C. auris* (Figure 6(d), Table S4), a large portion comprised cell membrane- and cell wall-associated genes. Namely, genes related to ergosterol biosynthesis (*ERG2, ERG6*) or chitin biosynthesis (*CHS3, CHS8*) and other related genes (*PMM1, FEN1, ECM17, WSC2*) were specifically up-regulated in *C. auris* (Figure 7(a)).

Species-specific differences also became obvious in terms of iron acquisition during whole-blood infection, as orthologous genes for ferric reductases (*FRP1, FRP2*), hemoglobin utilization (*RBT5, PGA7*), and siderophore transport (*SIT1*) were differentially regulated in *C. auris* and *C. albicans* during infection (Figure 7(a)).

While *C. auris* does not have orthologues of key *C. albicans* virulence genes, like *ECE1* or *PRA1* [12], *C. auris* expresses genes without orthologues in any of the other four analyzed *Candida* species. Of the 659 *C. auris*-specific genes analyzed in this study, over half of them (368 out of 659) were differentially expressed during at least one time point of infection (Table S7). Of note, 96 of the *C. auris*-specific up-regulated DEGs at 240 mpi were of unknown function, and others were associated with transmembrane transport (Figure S3). This includes genes potentially relevant for iron homeostasis and acquisition during blood infection, for example, B9J08_001548, B9J08_001547, and B9J08_001519. These genes have no clear orthologues but were found to be similar (by NCBI BLASTP analyses [84],) to genes encoding the siderophore transporter Sit1 (Figure 7(a)), and genes B9J08_000568 and B9J08_001951 were found to be similar to genes encoding the ferric reductase Fre3 and Csa1, a member of a hemoglobin-receptor family of *C. albicans*.

In summary, *C. auris* showed numerous transcriptional adaptations, including changes in membrane transport and the cell wall, likely enabling the fungus to cope with host-derived stresses and survive in the blood niche. Of note, this includes the up-regulation of known drug-resistance genes, which may contribute to drug resistance in a clinical setting.

### Candida auris *oxidative stress response during whole blood infection*

Blood-borne immune cells have to kill invading microbes and prevent microbial dissemination. One killing strategy of immune effector cells like neutrophils is the production of reactive oxygen species (ROS). This oxidative burst can be primed by cytokines such as TNF-α or chemokines such as IL-8. Increased expression of the corresponding genes (*cxcl8, tnf*) was indeed detected during the *C*. *auris*-blood infection (Figure 5). The activation of neutrophils was also indicated by the up-regulation of the early activation marker gene CD69 (> 5-fold up from 30 mpi on), and the gene encoding CD83 (> 12-fold up from 30 mpi on) both also known to be activated in neutrophils during *C. albicans* infections [39].

Contact to neutrophils, the most abundant immune cells in blood, causes fungal oxidative stress responses in *C. albicans* [68,85] and *C. glabrata* [86,87]. Consequently, we compared the expression of *C. auris* oxidative stress response genes during blood infection with the expression of correlated genes for other *Candida* species (Figure 7(a)).

The key genes encoding detoxifying enzymes of *C. albicans* are the superoxide dismutase genes *SOD4* and *SOD5*, which are all up-regulated during blood infection [53,68]. In contrast, the *C. auris* superoxide dismutase genes (*SOD1* and *SOD4*; no orthologue for *SOD5*) were only weakly up-regulated (Figure 7(a)). While the catalase gene *CAT1* and its orthologues were up-regulated in *C. albicans, C. glabrata, C. tropicalis* and *C. parapsilosis*, this was not observed in *C. auris* (Figure 7(a)). Furthermore, the glutathione system (including *GSH1, GPS2, GST1, GPX*s, and *GTT*s [85,88,89]) contributes to ROS detoxification in *C. albicans* and members of this gene set are up-regulated in *C. albicans, C. tropicalis*, and *C. parapsilosis*. In contrast, the corresponding orthologous genes (as far as they exist) are not up-regulated in *C. auris* or *C. glabrata* (Figure 7(a)), suggesting a minor role (at most) of the glutathione system in the oxidative stress response of these two species.

However, we observed that *PRX1* encoding the putative thioredoxin peroxidase was significantly up-regulated at early time points in *C. auris* (> 30-fold up) but not in *C. albicans* and *C. parapsilosis*, putatively contributing to ROS detoxification. A similar species-specific expression was visible for other components of the thioredoxin system (*TSA1, TRX1*, and *TRR1*) (Figure 7). Furthermore, the *C. auris* orthologues (B9J08_003622 and B9J08_000834) of *C. albicans* orf19.7085, which has been described to be oxidative stress-induced [90], were highly up-regulated during infection as were the orthologous genes in *C. tropicalis* and *C. parapsilosis* (Figure 7(a)).

Taken together, genes for ROS detoxifying enzymes and systems seem to be present in *C. auris* and other tested *Candida* species. However, their expression patterns during blood infections were species-specific, suggesting an individual strategy for *C. auris* to cope with immune cell-derived oxidative stress.

### Candida auris *interacts with human neutrophils*

**Figure 8.**
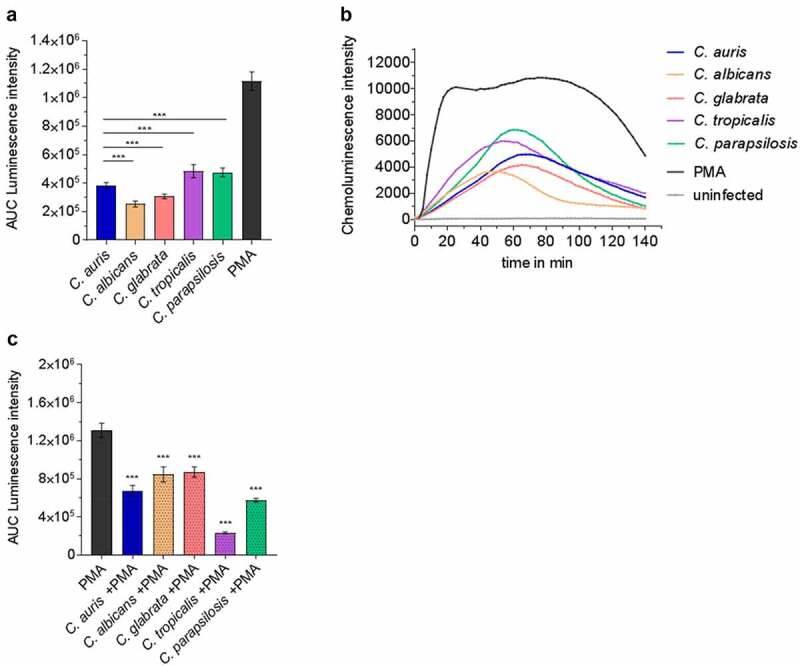
Human neutrophils recognize *C. auris in vitro* and respond with oxidative burst. (a) Human neutrophils were infected with different *Candida* strains at an MOI of 5. Phorbol 12-myristate 13-acetate (PMA) was used as a positive control. Results are shown as Area Under Curve (AUC) based on the ROS measurement presented in (b) n = 8, mean ± SD (B) ROS measurement over the course of co-incubation of different *Candida* strains with human neutrophils *in vitro*. MOI 5; n = 8; *** p ≤ 0.001 vs. *C. auris* (c) Human neutrophils were treated with PMA and in parallel infected with the indicated *Candida* spp. at an MOI of 10 to detect fungal ROS detoxification. Results are shown as AUC of the ROS measurement. n ≥ 7;  *** p ≤ 0.001 vs. PMA.

**Figure 9.**
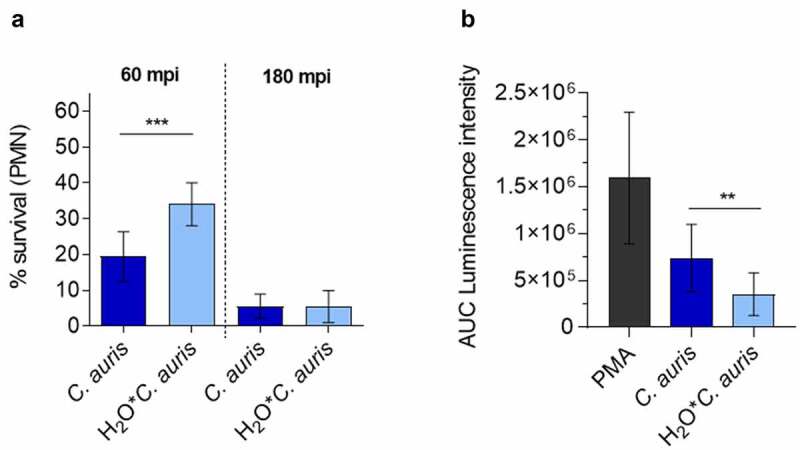
*C. auris* survival of neutrophil infection. (a) Survival of *C. auris* after 60 min or 180 min co-incubation with human neutrophils *in vitro*, determined by CFU quantification. n = 6, mean ± SD; *** p≤ 0.001 (b) Induction of ROS in human neutrophils after co-incubation with *C. auris* compared to *C. auris* pre-starved in water (H_2_O*C. auris) over 6 days. n = 6, mean ± SD; ** p≤ 0.005 .

Since neutrophils and their oxidative burst are key players during *Candida* bloodstream infections (Figure 3(c) [53,68]), we investigated the direct interaction of *C. auris* with neutrophils in comparison to the other *Candida* species. Neutrophils clearly showed ROS production in response to *C. auris* and all tested *Candida* species (Figure 8(a,b)). However, as suggested by the transcriptional profiles, all the investigated *Candida* species were able to detoxify ROS produced by PMA-stimulated neutrophils (Figure 8(c) [91]). We then analyzed the killing of *C. auris* by neutrophils. Only 15% of *C. auris* cells survived after 60 mpi and the overall viability of fungal cells were further decreased to 2% 180 mpi, underlining the key role of neutrophils in the blood against *C. auris* infections (Figure 9(a)).

### *Effects of the environmental preconditions on the survival rate of* C.auris

Although we did not observe greater resistance of *C.auris* to killing in whole blood or by human neutrophils compared to other clinically relevant *Candida* species, it is the *Candida* species of major concern for hospital outbreaks. We next questioned whether the high-stress resistance and the high capacity of *C.auris* to survive harsh environmental conditions and nutrient limitation (Figures 1, 2) may be relevant for the transfer from the environment to a patient. To analyze the influence of previous environmental conditioning on interactions with blood cells, we starved *C. auris* in water and subsequently determined fungal survival after co-incubation with human blood-derived neutrophils *in vitro*. We observed a significantly increased survival rate at 60 mpi after pre-starvation in water (Figure 9 (a)). In contrast, pre-starvation did not affect survival rates at the later stages of infections. Potentially, growth under harsh environmental conditions changes fungal cell properties, which may dampen antifungal activity by host cells. In fact, ROS production by neutrophils was significantly less pronounced after infection with pre-starved *C. auris* cells compared to standard conditions (Figure 9(b)).

These data suggest that the environmental conditions *C.auris* faces prior to infection can modulate the initial interaction between human neutrophils and early bloodstream fungal survival.

## Discussion

Infections with *Candida* species are considered a global public health threat, especially in the nosocomial setting [92,93]. While *C. albicans* is still the most common cause of life-threatening candidiasis and the best-studied *Candida* species, the incidence of and interest in non-*albicans* species such as *C. tropicalis, C. parapsilosis, C. glabrata*, and *C. auris* is increasing [94]. Lately, *C. auris* infections became of particular public concern due to outbreaks in health care settings in different countries, the unusual high intrinsic antifungal resistance of this fungus, and by its spread in specialized COVID-19 ICUs during the current pandemic [95,96]. A better understanding of the pathogenicity of *C. auris* is therefore vital.

In our study, we shed light on the interplay between *C. auris* and human blood cells during *ex vivo* blood infection by characterizing cellular interactions with blood cells, the immune response and the transcriptome of both host and fungal cells using dual-species RNA-Seq. Our study reveals a human response to *C. auris* infection that is dominated by early immune system activation, and which is comparable to infections with other *Candida* species. On the fungal side, the transcriptional changes of *C. auris* during whole-blood infection were clearly different in comparison to other medically important *Candida* species, indicating a specific response of *C. auris* to blood exposure that does not resemble known *Candida* species.

While *C. auris* does not appear to be more pathogenic than other *Candida* species in our model, it has caused hospital outbreaks with severe nosocomial transmission [24,31,95,97,98]. Our analyses of *C. auris’* resistance against stress and harsh *in vitro* conditions showed a remarkable ability of this fungus to survive in the environment for a long time. This provides a possible explanation for the scale of nosocomial infections, as *C. auris* may survive better on many different hospital surfaces than other *Candida* species. Importantly, the same characteristics may not only facilitate nosocomial survival and persistence, but also resistance to host-derived stressors during the early stages of infection.

### *Human responses to* C. auris *blood infection*

During blood infection, the vast majority of infecting *C. auris* cells were associated with neutrophils, similar to *C. albicans* and *C. glabrata*, underlining the importance of this interaction in fungal blood infections in general (Figure 3(c) [53,99]). Furthermore, some fungal cells associated with monocytes (Figure 3(c) [42,53,100]).

The human transcriptional response is likely derived predominantly from these blood-resident immune cells. These immune cells (neutrophils, monocytes) have a preformed repertoire of immune mediators, explaining an initially limited transcriptional response to infections with any of the five *Candida* species, clearly increasing after 60 mpi (Figure 4(b), Figure S2A [53]). Our transcriptional data indicate a recognition of *C. auris* by these cell types *via* PRRs, including the C-type lectin receptors (CLRs) MRC1 and CLEC5a, the complement receptor CR3, and the Toll-like receptor TLR2 (Figure 5). This activates MAPK-related signaling, NF-κB-signaling, and EGR regulators that lead to pro-inflammatory activities, including the expression and secretion of cytokines like IL-1α, IL-1β, IL-6, IL-13, and TNF-α, as well as chemokines and other anti-fungal responses (Figure 5). Similarly, CLR-associated TNF-α, IL-6, IL-1β, and chemokine expression was reported by isolated PBMCs in response to *C. auris* [37]. In our blood model, comparison of *C. auris* infections with previous data on *Candida* species demonstrated that the transcriptional response on the host side was largely conserved and mainly dependent on the time point of infection rather than on the infecting species – in line with our previous four-species observations (Figure 4(a) [53,54,68]). The common human response even extends to infections with *C. albicans* cells, although these transition to hyphae during blood infection [53,54], while the other four species, including *C. auris*, remain in the yeast morphology (Figure 3 [53]). Niemiec *et al*. described a morphology-independent neutrophil response toward *C. albicans* [39], which resembled the response to infections with *C. auris* in our study, including the up-regulation of *PTX3, NLRP3, EGR1-3, TNF*, and chemokine genes (Figure 5). An efficient early neutrophil activation by non-filamentous *C. albicans* strains (when components of the complement system are present) has been reported earlier [101], which is also the case in our blood model.

On the host side, we therefore show that the antifungal defense is effective in fungal killing, dominated by pro-inflammatory responses, and largely conserved against several *Candida* species.

### C. auris *responses to human blood*

In line with the human immune response toward *Candida* cells, the majority of *C. auris* cells are cleared in our blood infection model over 240 min (Figure 3(a)). However, a small fungal subpopulation survived, and in the *in vivo* situation an escape from the bloodstream would be expected within this timeframe [102,103]. Therefore, our data provide experimental evidence that a portion of *C. auris* cells can survive long enough to escape from the bloodstream as a prerequisite to colonize organs. Our data indicate that *C. auris* is in fact able to proliferate in murine kidney and liver homogenates, as well as in organ-simulating medium, comparable to, or at even higher rates than other *Candida* spp. (Figure S4).

Transcriptional profiling was used to elucidate the contribution of *C. auris* genes and potential virulence factors to adaptation and survival in blood. However, gene functions have so far been mainly ascribed to orthologue information, rather than functional characterization in *C. auris* itself. Nevertheless, the genome of the *C. auris* strain B8441 has been sequenced and annotated by comparative genomic analyses, which showed conservation of, amongst others, genes associated with virulence within the *Candida* clade [12,32,94].

At the transcriptional level, metabolic adaptations to the bloodstream environment were observed for *C. auris* as well as for other *Candida* species. However, the regulation of genes in catabolic and anabolic pathways differed (Figure 7). For example, *C. tropicalis* and *C. parapsilosis* up-regulated β-oxidation, and *C. albicans, C. tropicalis*, and *C. parapsilosis* up-regulated glyoxylate cycle genes, which was not the case for the orthologous *C. auris* genes throughout the course of infection (Figure 7). Contact to neutrophils was also previously described to induce amino acid synthesis, e.g. the methionine biosynthesis pathway in *C. albicans* [85,104]. We found genes of this pathway (*MET1, MET10*) up-regulated in *C. albicans* and *C. auris* at early stages of blood infection, while this was not seen in *C. parapsilosis* (Figure 7 [53]). As amino acids are present in blood, they might be acquired *via* transporters to serve as carbon and nitrogen sources [105]. Especially in the *C. auris* genome the number of transporter genes, like the oligopeptide transporter family (*OPT* genes) that enable the uptake of small peptides, is expanded [12]. The up-regulation of several *C. auris* amino acid transporter genes (*GAP4, OPT1*, B9J08_004537 (*OPT2*), *PUT1*; Figure 7), suggests that amino acids are indeed imported as a nutrient source during blood infection. Overall, these data indicate the use of different carbon sources by the different *Candida* species during blood infection. This may be explained by variable nutrient acquisition and metabolic strategies evolved in the individual host- or environment-associated natural reservoirs of each fungus [105].

Further interspecies differences are expected due to the fact that distinct virulence traits like the yeast-to-hypha transition and the peptide toxin candidalysin of *C. albicans* are not present in *C. auris* [12]. Filamentation contributes to *C. albicans* survival in blood [53] and might account for the moderately higher survival of *C. albicans* compared to *C. auris* (Figure 3). Other genes encoding virulence-associated factors of *C. albicans*, like secreted proteases (Saps) and lipases (Lips) have orthologues in the *C. auris* genome [12,94]. The *LIP10* and the *SAP9* orthologues of *C. auris* as well as two other genes with similarity to *SAP9* (B9J08_003911, B9J08_005335) were significantly up-regulated during incubation in blood (Figure 7) and may contribute to the interaction with blood cells. For example, *C. albicans* Sap9 has been shown to be required for neutrophil activation including chemotaxis, ROS formation, and NETosis [106,107], and its *C. auris* counterparts may have similar roles.

Furthermore, *C. auris* harbors an expanded set of transporter genes, including several members of the ABC and MFS gene families [12,81,108]. Interestingly, genes of the expanded MFS class, known for their role in antifungal resistance [12,94], seem to be relevant during *C. auris* blood infection as *MDR1*, but also *TPO3* and related genes (B9J08_000368, B9J08_003036), were highly up-regulated in our study (Figure 7). This regulation pattern is unique to *C. auris* compared to the other four analyzed *Candida* species, suggesting a specific role of these transporters during *C. auris* during blood infection. The physiological role of MFS-MDR transporters might include the transport of lipids, ions, or other small metabolites during adaptations to the (host) environment [109]. In *C. lusitaniae*, Mdr1 has been shown to confer resistance to bacterial and host products like methylglyoxal or hydrogen peroxide [110,111] and *MDR1* expression in *C. albicans* was found under oxidative stress conditions [109,112]. Moreover, the ABC transporter Afr1 of *Cryptococcus neoformans* has been shown to be relevant for virulence by mediating protective effects against phagocytes [113]. While these studies suggest that transmembrane transporters can contribute to fungal fitness in challenging environments, the up-regulation of members of MFS transporter genes like *MDR1* in *C. auris* during blood infection may at least partially contribute to *C. auris* azole resistance in patients. This up-regulation of genes involved in counteracting azoles in the blood niche might prepare *C. auris* for subsequent antifungal therapy, contributing to clinical failures to clear this fungus with this class of drugs during infections.

Another gene family that is expanded in *C. auris* comprises *SIT* (siderophore transporter) genes, related to iron uptake and homeostasis [12]. The up-regulation of *SIT1* and related genes (B9J08_001519, B9J08_001548, B9J08_001547) during blood infection highlights the relevance of these processes for *C. auris* in an environment which is virtually free of unbound iron ions (Figure 7). An association of the previously mentioned expanded *C. auris* gene families with host interactions and fungal pathogenicity is further in line with earlier observations on gene expansion in *C. albicans* [114].

Variations in the cell surface structures between different species likely influence immune recognition and response [52]. Unique mannan structures as well as different chitin levels of the *C. auris* cell wall compared to other *Candida* species have been described [37,52,115]. During blood infection, expression of genes associated with cell wall biogenesis and remodeling was different for *C. auris* in comparison to the other *Candida* species (Figure 7). Especially genes related to ergosterol and chitin synthesis, as well as *PGA* genes, were primarily up-regulated in *C. auris*. This suggests that *C. auris* modulates its cell wall in response to the host differently than other *Candida* spp., which in turn might contribute to the few *C. auris*-specific transcriptional changes in the host response observed in this study.

Taken together, *C. auris*’ transcriptional adaptations to the blood niche especially relate to metabolism, cell wall remodeling, and acquisition of micronutrients – processes that are also regulated by other *Candida* species during blood infection. However, much of the transcriptional patterns and kinetics were species-specific and suggest an independent strategy for *C. auris* to deal with the blood environment. Of note, this includes the up-regulation of genes, especially encoding transporters, known to be associated with azole resistance.

### *Neutrophils as the key player during* C. auris *blood infections*

The interaction of *C. albicans* with blood cells has been investigated with isolated immune cells as well as in blood models [40,41,53,68]. Fradin *et al*. demonstrated that the transcriptional profile of *C. albicans* in blood is dominated by transcriptional responses to neutrophils [68]. The generation of reactive oxygen species (ROS) is a central defense mechanism of neutrophils against fungal pathogens [40,41,116,117]. The generation of ROS by neutrophils was also seen after contact to *C. auris* (Figure 8). It appeared slightly delayed compared to *C. albicans* (Figure 8(b)), which is known to be quickly recognized and attacked by neutrophils [42], but seemed comparable to the other *Candida* spp.

In response, fungal cells express a plethora of genes to counteract oxidative stress [68]. However, the individual oxidative stress adaptations differed (Figure 7). This can be explained in part by a different genetic repertoire, for example, *C. albicans* has an evolutionarily expanded set of superoxide dismutase (*SOD*) genes [85]. *C. auris* does not have a orthologue of *SOD5* but does possess a *SOD4* orthologue which was up-regulated during blood infection, suggesting a contribution of at least one Sod to detoxify O_2_^−^ in *C. auris* (Figure 7 [118]). *C. auris* orthologous genes of other antioxidant mechanisms like catalases (*CAT1)* or the glutathione/glutaredoxin (*GSH, GPX, GST*) system [85] were not up-regulated during blood infection, while they are used by *C. albicans* and *C. glabrata* under oxidative stress conditions [67]. However, the thiol-specific peroxidase Tsa1 of the thioredoxin system (*TSA1, TRX1, TRR1*), known to protect *C. albicans* against ROS [85], also seems to be relevant for *C. auris* during oxidative stress in blood, as the orthologous gene was significantly up-regulated (Figure 7). Furthermore, the putative peroxidase gene *PRX1* and numerous genes encoding heat shock proteins and enolases, shown to be involved in fungal oxidative stress response [119], showed high mRNA copy numbers in *C. auris* during blood infection (Figure 7). Accordingly, the neutrophils’ ROS production can be detoxified by *C. auris* as well as the other four *Candida* species (Figure 8).

In our study, substantial killing of *C. auris* by isolated human neutrophils was observed 60 mpi and further increased at 180 mpi, in line with the induction of ROS (Figure 9, Figure 8). In contrast, Johnson *et al*. reported inefficient killing of *C. auris* by human neutrophils, which is concordant with reduced ROS generation compared to *C. albicans* [36]. This may partially be explained by the use of different *C. auris* strains or more likely by the fact that infections in our study include opsonization with human serum, which was shown to be critical for neutrophil activation in our experimental settings (Figure S5). The crucial role of opsonization for efficient host cell response and killing of *Candida* species by phagocytes – as seen for *C. auris*-neutrophil interactions in our study – is in agreement with previous studies [120–122], including *C. auris*-infected PBMCs [37]. Along the same line, ROS production by neutrophils was observed with serum-opsonized, heat-killed *C. auris* cells (Figure S5 [37]) and Bruno *et al*. demonstrated myeloperoxidase (MPO) production by neutrophils in *C. auris-*infected mice, demonstrating *in vivo* activation of neutrophils during *C. auris* infection [37].

Combined transcriptional and cellular observations of *C. auris* during blood infection and interactions with isolated neutrophils demonstrated that *C. auris* experiences oxidative stress. Despite oxidative stress response and high H_2_O_2_-resistance, *C. auris* is efficiently killed in blood and by neutrophils *in vitro*, suggesting an effective combination of oxidative burst with other anti-microbial mechanisms in these *in vitro* and *ex vivo* settings and thus potentially also *in vivo*.

### C. auris *stress resistance impacts environmental survival and virulence*

*C. auris* infections are often reported as outbreaks in health-care settings, which seem to be linked to fungal persistence in the hospital environment [23,95] and intra- or inter-hospital *C. auris* transmission [9,24,97,123,124]. Nosocomial transmission is well known for *C. parapsilosis*, while infections with *C. albicans, C. glabrata* or *C. tropicalis* are thought to mainly originate from endogenous fungal populations [125–128].

In our study, long-term incubation of *Candida* cells in water (Figure 2(a,b)) lacking any nutrients or on plastic surfaces, similar to material found in hospital settings (Figure 2(c)) revealed a remarkable ability of *C. auris* to survive harsh environmental conditions over several days or even weeks – in contrast to all other tested *Candida* species, except the other majorly hospital-environmental species *C. parapsilosis* [129], which is in agreement with previous studies on *C. auris* and *C. parapsilosis* [23,130,131]. Thus, we have shown and confirmed that *C. auris* can survive and persist for long periods outside of the human host, and that nosocomial *C. auris* infections can therefore originate from environmental sources, such as contaminated medical devices or hands of health care workers, without prior colonization of the infected host [95,126]. This increases the risk of transmission in healthcare settings [24,108,132] and explains why hospital outbreaks are described for *C. auris* and *C. parapsilosis* rather than for the other *Candida* species [16,17,131].

We propose that features, which increase environmental survival of *C. auris*, including resistance to environmental stressors, like UV light, dryness, or salt, might also have effects on the interaction with the human host and contribute to *C. auris* virulence. We therefore analyzed the interaction of *C. auris* cells pre-grown under harsh environmental conditions with human neutrophils (Figure 9). In fact, these experiments suggest that environmental conditions can significantly influence subsequent infections with immune cells at early stages. Such impact on *C. auris*-host interactions after environmental starvation or other stressful conditions might be explained by differences in basal gene expression, storage levels of essential micronutrients or cell surface characteristics. The latter would likely influence recognition and responses by the innate immune system, as demonstrated for *C. albicans* in response to pH or defined nutrient sources [133–135]. Finally, it is possible that such pre-conditioned *C. auris* cells may be more suitable to colonize the skin [28].

We conclude that the remarkable ability of *C. auris* to resist environmental stressors and to adapt to harsh long-term conditions can impact environmental survival, the existence of hospital pools of viable and infectious cells and the initial phase of infections.

## Supplementary Material

Supplemental Material

## Data Availability

The RNA-Seq data that support the findings of this study are openly available in NCBI’s Gene Expression Omnibus under the GEO record GSE179000and GSE114180. https://www.ncbi.nlm.nih.gov/geo/
